# Phylogenetic corrections and higher-order sequence statistics in protein families: Potts vs multiple sequence alignment transformer machine learning models

**DOI:** 10.1103/g5cx-1vhj

**Published:** 2025-10-20

**Authors:** Kisan Khatri, Ronald M. Levy, Allan Haldane

**Affiliations:** 1 Department of Physics and Center for Biophysics and Computational Biology, Temple University, Philadelphia, Pennsylvania 19122, USA; 2 Department of Chemistry and Center for Biophysics and Computational Biology, Temple University, Philadelphia, Pennsylvania 19122, USA

## Abstract

Recent generative machine learning models applied to protein multiple sequence alignment (MSA) datasets include simple and interpretable physics-based Potts covariation models and other machine learning models such as MSA Transformer (MSA-T). The best models accurately reproduce MSA statistics induced by the biophysical constraints within proteins, raising the question of which functional forms best model the underlying physics. The Potts model is usually specified by an effective potential including pairwise residue-residue interaction terms, but it has been suggested that MSA-T can capture the effects induced by effective potentials that include more than pairwise interactions and implicitly account for phylogenetic structure in the MSA. Here, we compare the ability of the Potts model and MSA-T to reconstruct higher-order sequence statistics reflecting complex biophysical sequence constraints. We show how the model performance depends greatly on the treatment of phylogenetic relationships between the sequences, which can induce nonbiophysical mutational covariation in MSAs. When using explicit corrections for phylogenetic dependencies, we find that the Potts model outperforms MSA-T in detecting epistatic interactions of biophysical origin. Furthermore, the sequences generated by the models we studied here are predicted to fold into nativelike structures, as indicated by AlphaFold predictions.

## I. INTRODUCTION

Machine learning models have made great strides in predicting the functional and structural properties of proteins based on large protein sequence datasets, including the subclass of generative protein sequence models (GPSMs) that learn an underlying sequence probability distribution *P*(*S*) from a multiple sequence alignment (MSA) to generate new synthetic protein sequences *S*. To function well, GPSMs must capture covarying amino acid patterns in the MSA that implicitly encode information about physical constraints on proteins, enabling the design and detection of hidden protein properties from sequence data [1,2]. This raises the question of which GPSM functional form best describes protein biophysics, which best distinguishes the different causes of covariation, and how to measure this.

**FIG. 1.**
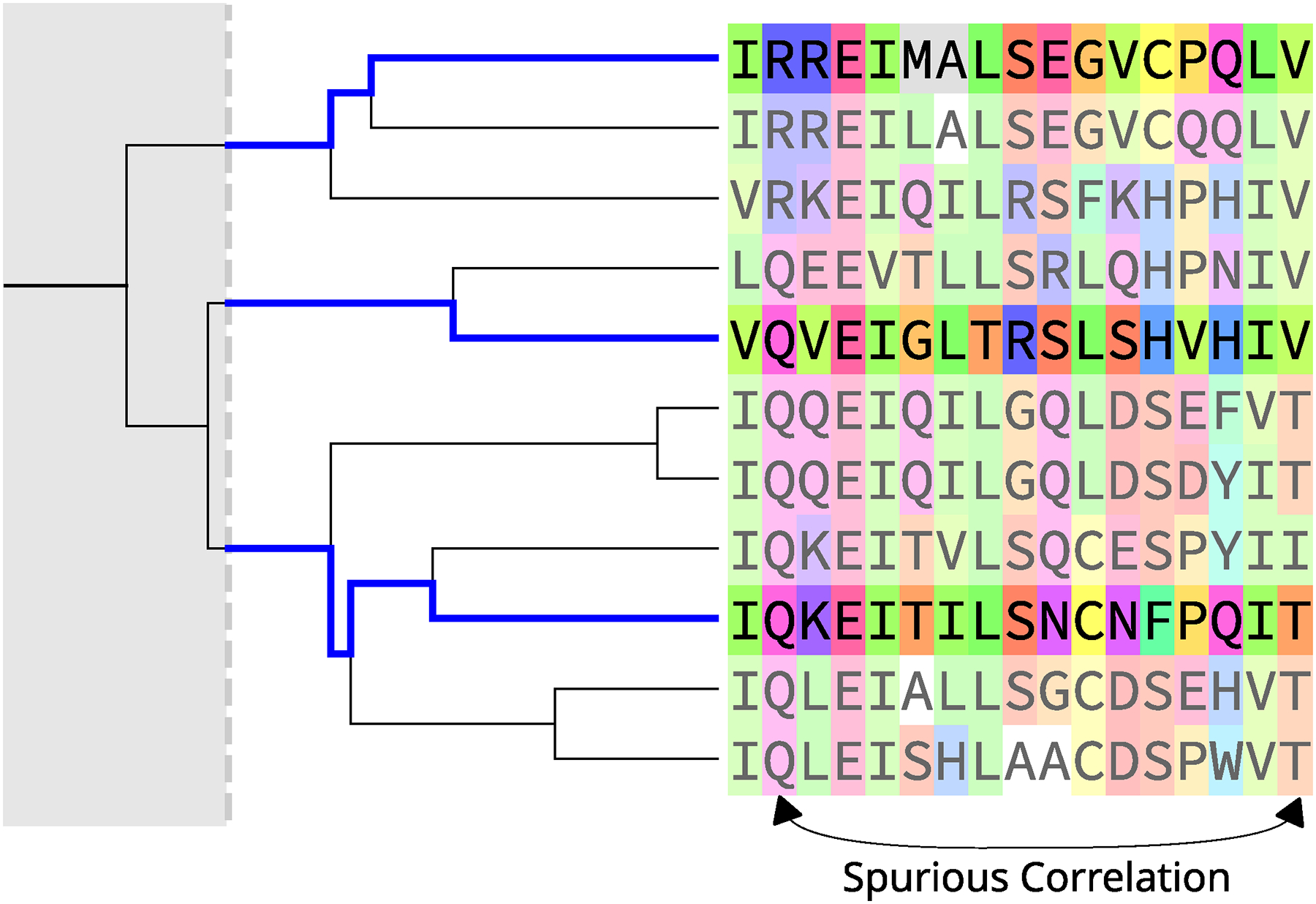
Phylogenetic relationships between sequences in an MSA result in a spurious mutational correlation due to common ancestry, for R/V and Q/T combinations at the illustrated column pair. Sequence pairs greater than an identity cutoff (diverging to the left of the gray dotted line) approximate i.i.d. samples, so that identity filtering to retain three sequences labeled in blue gives an unbiased sample showing no correlation with equal frequency of R/V, Q/V, and Q/T combinations.

Mutational covariation in protein sequences provides a highly informative statistical signal that accurate GPSMs must capture. Covariation arises from multiple factors, including the following [3]: (1) High-fitness sequence motifs and epistasis (mutational cooperativity) underlying biophysical function lead to compensatory mutation pairing patterns between residues. (2) Phylogentic relationships due to recent speciation or gene duplication can distort covariation patterns, causing “excess” counts of patterns from evolutionarily related sequence clusters as illustrated in Fig. 1. Statistical noise due to the limited number of available distinct natural sequences used during model inference introduces finite-sampling statistical variation in estimated covariation and in GPSM accuracy. The latter two can be considered nuisance factors in uncovering the underlying biophysics driving the first factor of fitness-induced covariation. These covariation signals are measured from MSAs of individual protein families, which consist of homologous proteins with shared function and overall structure.

We examine how two leading GPSMs in recent focus distinguish these effects, the Potts model [1,4–9] and the MSA Transformer (MSA-T) [10,11], with careful control for how phylogenetic clusters affect GPSM performance. We suggest that the most reliable metrics to measure GPSM performance use statistical characteristics of individual protein families designed to exclude the biasing effects of phylogenetic relationships between sequences that do not originate from the fundamental biophysical properties of the protein family. We find that when the impact of phylogenetic relationships is accounted for, the Potts model better captures one such set of statistics, the higher-order MSA statistics, referring to the covariation patterns involving more than two amino acids. We expect these complex mutational dependencies to reflect epistatic networks underlying protein function, structure, and evolution [12], forming a more challenging test than capturing simple pairwise covariation.

## II. GPSM ARCHITECTURES

Despite their quite different architectures and complexity, the Potts model and MSA-T exhibit fundamental commonalities in their design to account for covariation patterns [13]. The Potts model is a statistical physics model inspired by spin glasses that is increasingly being adapted as a generative modeling tool in machine learning to capture sequence variability within a single protein family. A Potts model trained on protein family *F* has probability distribution $P\left(S|\theta_{F}\right)\propto \text{exp}\left(-\sum_{i<j}J_{s_{i}s_{j}}^{ij}\right)$ for sequences *S* to evolve, with characters *s*_*i*_ at position *i*, and pairwise “coupling” parameters $\theta_{F}=\left\{J_{\alpha \beta }^{ij}\right\}$ measuring the favorability of having residues *α, β* at positions *i, j* in a sequence. It models complex higher-order correlations through networks of pairwise interactions. In contrast, MSA-T is a deep learning [14] masked language neural network attention model [15] trained only once on MSAs of all available protein families simultaneously [10] with about 10^4^ times more parameters, recently used for protein structure and evolution analyses [16–22]. The Potts model has been found to have superior generative accuracy to some other GPSMs, including variational autoencoders and site-independent models [12], but a generative method subsequently developed for MSA-T has been reported to better reproduce higher-order statistics [23], though its parameters lack clear physical interpretation and the reasons for this result are ambiguous.

## III. EFFECT OF PHLYOGENETIC RELATIONSHIPS ON GPSM EVALUATION

GPSMs must distinguish the causes of covariation, and in the case of the Potts model, an identity filtering procedure is critical. The Potts model is trained to reproduce the site statistics of a training MSA from the protein family, assuming each sequence is an independent and identically distributed (i.i.d.) sample. To be i.i.d., the sequences can be envisioned to have evolved from a distant ancestral sequence under a common fitness function and mutational process encoded in *P*(*S* | *θ*_*F*_), and enough time must have passed for any statistical correlation with each other to be effectively nil due to mutational saturation. Phylogenetic relationships violate the *independence* assumption, as clusters of orthologs and other closely evolutionarily related sequences are explicitly non-i.i.d. In standard Potts methodology, the related sequences are filtered to eliminate phylogenetic redundancy.

In contrast, MSA-T accounts for phylogenetic relationships through a columnwise attention layer [11,21], and is not explicitly trained on MSA statistics but rather on a masked prediction task insensitive to phylogenetic structure, in predicting character states in input MSAs from all protein families at once, which were randomly masked. A method has been proposed to use MSA-T in a generative fashion for a single protein family by repeatedly masking and resampling an input MSA of that family to produce novel sequences [23]. The MSA-T probability distribution for this generative method is *P*(*S* | *θ, M*_*F*_*, p*) for generating sequence *S*, and depends the model’s pretrained parameters *θ*, an input MSA *M*_*F*_ for a protein family *F*, and a masking frequency parameter *p*. Unlike the Potts model that is trained separately for each protein family, MSA-T is trained only once on all protein families, and the generation of new sequences does not involve any additional training but instead iterated prediction given an initial MSA of a given family.

One goal of this study is to control for how phylogenetic clusters affect GPSM performance. After filtering, the i.i.d. MSA will have covariation signals that exclude biases due to clustering and so are presumably biophysical in origin, and should be reproducible in arbitrary subsets of the MSA. This provides a foundation for determining which GPSMs best capture biophysical constraints.

## IV. STATISTICAL METHODOLOGY

**FIG. 2.**
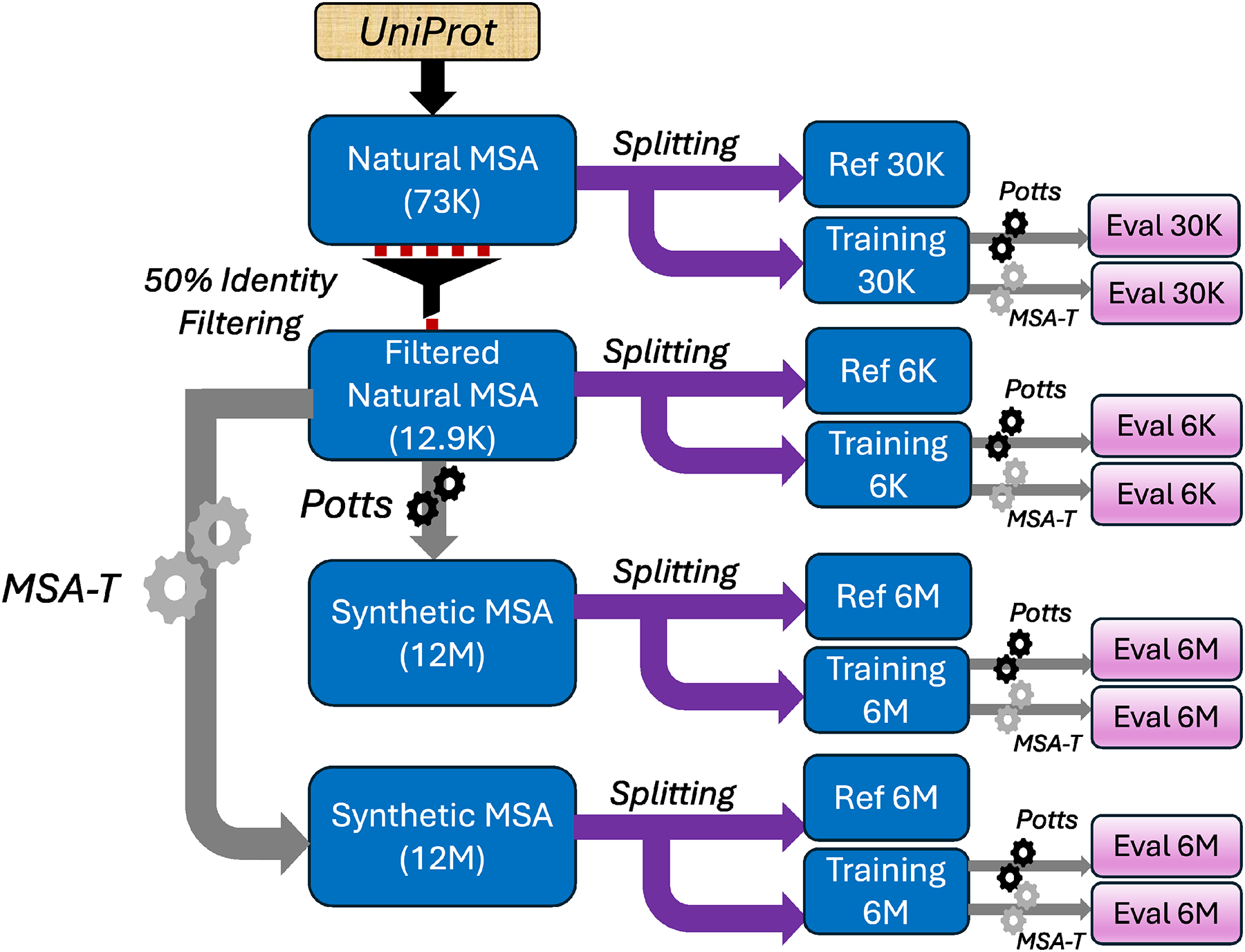
Overview of statistical tests carried out using the Potts model and MSA-T to isolate different forms of statistical error. Boxes represent MSAs with different amounts of sequences shown for the RR-domain family, which are then filtered, split, or used to train and generate from the GPSMs (arrows). Black gears represent the Potts model and light gray gears represent MSA-T. Our tests measure the statistical difference between the “evaluation” MSAs and the corresponding “reference” MSAs.

We perform tests of GPSM performance with and without accounting for phylogeny as outlined in Fig. 2. We generate sequences from MSA-T using the default method of Ref. [23] using precomputed parameters *θ*, and train the Potts model using the high-accuracy Mi3-GPU method [24]. We also test a very simple GPSM, the Independent model, with distribution $P(S)=\prod_{i}f_{s_{i}}^{i}$, where $f_{\alpha }^{i}$ are single-site frequencies for amino acid *α* at position *i* found in the training MSA, which is unable to accurately capture even the pairwise amino-acid frequencies $f_{\alpha \beta }^{ij}$ of the training MSA. Given various training MSA data described in subsequent sections, we generate new MSA datasets from each GPSM and then evaluate GPSM performance by comparison of the generated “evaluation” MSA to a “reference” MSA using a metric *r*_20_, which measures higher-order covariation and provides a more stringent test of GPSM accuracy than pairwise covariation statistics or point-mutant effects [12]. The training, reference, and evaluation MSAs have the same number of sequences. These tests are designed to isolate the effects of fitness, phylogeny, and statistical noise, and to control statistical errors including model specification, out-of-sample, and estimation errors [12], such as by splitting each MSA dataset into training and reference MSAs to ensure that no sequences used to train the model are used in its evaluation. We examine two protein families: RR domain (PF00072), which was previously studied to test MSA-T predictions of higher-order sequence statistics [23], and protein kinase (PF00069) [12]. The details of the methodology are provided in the Appendix.

To characterize these patterns, we compute the frequencies of noncontiguous amino acid “words” of length *n*, or higher-order marginals (HOMs) in both reference and model-generated evaluation MSAs, as in Ref. [12]. For each HOM length, we randomly select 3000 position sets, extract the top 20 most frequent words, and calculate the Pearson correlation (*r*) between their frequencies for each position set in those two MSAs. The averaged *r* over all sets is defined as *r*_20_ score, a metric that evaluates the model’s ability to reproduce higher-order sequence statistics and model complex chains and networks of interactions within protein [12]. This statistics for *n* = 2 measures how well the model reproduces the pairwise site statistics, and in the Supplemental Material [25], we illustrate the model performance for predicting pairwise mutational covariation (Fig. S1). As a reference point, we also compute a “null” result that represents the maximum attainable value for the *r*_20_ metric for a well-specified model subject only to finite-sampling limitations in model evaluation, in other words for an idealized GPSM with no specification or out-of-sample error. It is computed by measuring *r*_20_ for two independent MSAs drawn from the same probability distribution, the training MSA and reference MSA [12]. This provides a baseline for quantifying how accurately GPSMs model the target distribution, and deviations from the null result indicate imperfect modeling.

To perform identity filtering of MSAs, we iteratively find pairs of sequences in the MSA that are more than 50% similar and randomly drop one of the two. It is well known that proteins sharing the same fold often have as low as 8% sequence identity [26,27], and in the Pfam database [28], one observes that most protein families have between 10% and 50% average sequence identity, so that pairs with >50% identity suggest recent ancestry and non-i.i.d. sequences. This largely eliminates the influence of phylogenetic clusters on MSA statistics so that most covariation in the training MSA is biophysically induced. UniProt contains approximately 73K sequences for the RR-domain and 292K sequences for the protein-kinase families. After 50% identity filtering, the RR-domain dataset reduces to 12.9K sequences, which are further split into 6K for reference and training. For the protein-kinase family, the 292K sequences are filtered down to 20K. We also performed our analyses using MSAs filtered at 60% and 90% cutoffs to test whether the results were sensitive to cutoff, finding qualitatively they were not.

## V. THE POTTS MODEL OUTPERFORMS MSA-T AFTER PHYLOGENETIC PREPROCESSING

**FIG. 3.**
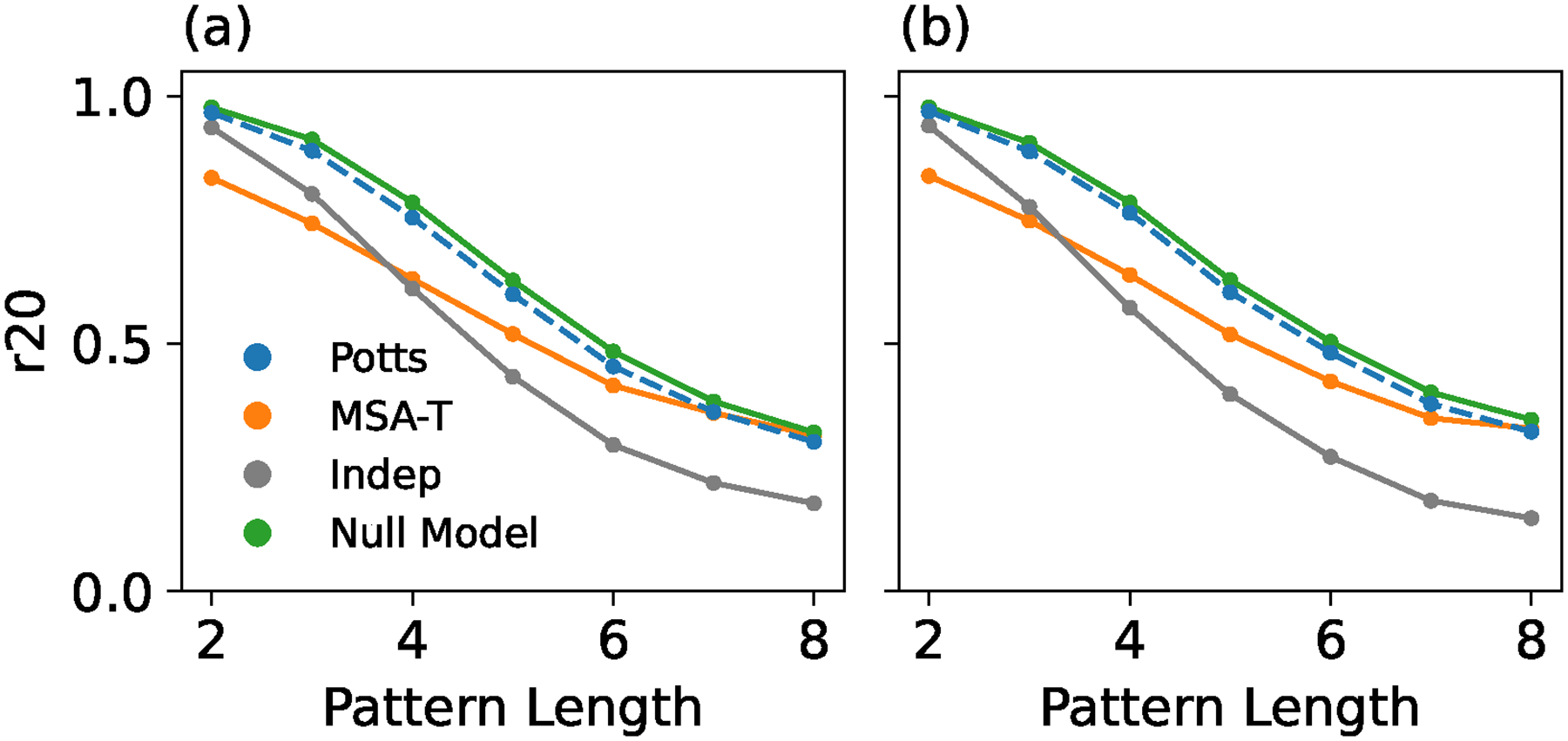
“Natural” GPSM performance test in which the training and reference MSAs are natural sequences filtered by sequence identity to eliminate phylogenetic redundancy, and evaluated using the *r*_20_ metric for (a) RR domain (MSAs of 6K) and (b) kinase protein (MSAs of 10K).

We first tested the performance of the model after filtering the training and reference MSAs, corresponding to the middle section of Fig. 2. In Figs. 3(a) (RR domain) and 3(b) (kinase), we show that the Potts model outperforms MSA-T in this test.

The *r*_20_ metric is lower at higher orders because of greater finite sampling error when measuring the smaller frequencies at these orders, and not because of reduced model accuracy [12]. This effect is apparent in the “null” result described above, which represents the upper bound to the *r*_20_ scores possible, given finite sample size. The Potts model results closely match the “null” result, unlike MSA-T.

**FIG. 4.**
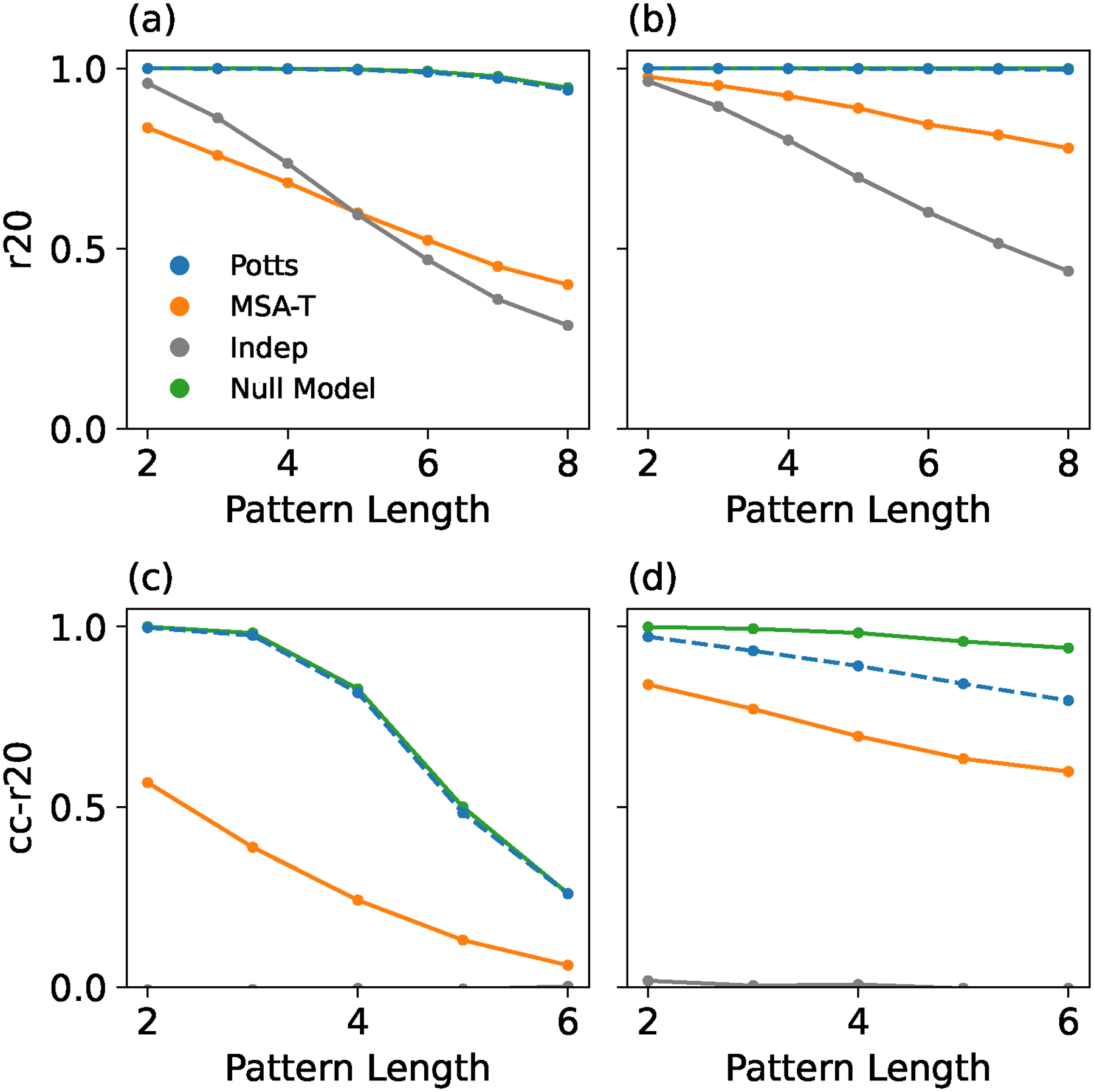
“Synthetic” GPSM performance test for the RR domain in which large (6M) training and reference MSAs are produced by an initial GPSM, which is a Potts model in panels (a) and (c), and MSA-T in panels (b) and (d). The *r*_20_ metric is used in panels (a) and (b), and a cc-*r*_20_ metric in panels (c) and (d).

The filtered natural MSAs have limited numbers of sequences (12.9K for RR domain), which causes significant finite-sampling error at high orders of marginal. To bypass this limitation, we conducted a “synthetic” test with large training and reference MSAs of 6M sequences. Here, we first trained an initial Potts model on filtered natural MSA and generated two synthetic 6M MSAs from it to serve as reference and training MSAs in the next step. A new Potts, MSA-T (as implemented in Ref. [23]), and an Independent model were trained on this synthetic i.i.d. training dataset. The result shown in Fig. 4(a) supports that the Potts model outperforms MSA-T when the input sequences are i.i.d. even at higher orders of marginal, which are better probed in this test. Interestingly, MSA-T performed worse than a site-independent model for low orders but slightly outperformed it at higher orders in the case of a synthetic Potts process. This synthetic test might not accurately represent how GPSMs would perform on natural datasets because the reference MSA was generated by the Potts model itself, and the refit Potts model should have zero specification error by construction. To address this, we performed another test in which the training and reference MSAs were generated by MSA-T from the natural dataset, showing in Fig. 4(b) that the Potts model still outperforms MSA-T. If MSA-T captures biophysical constraints in natural MSAs, which the Potts model cannot, we would instead expect lower Potts model performance. Interestingly, MSA-T is unable to reproduce the HOMs when trained on its own generated MSAs, as the *r*_20_ metric at higher order is lower than both the null expectation and the Potts result. We investigated this by testing the alternative generation algorithms presented in Ref. [23], as well as changing the acceptance rate *p* and other variations, but always found qualitatively similar results suggesting a general limitation of MSA-T.

An even more stringent test of the GPSMs is to measure a “connected correlation *r*_20_” (cc-*r*_20_) metric that compares connected correlations, which are higher-order generalizations of the pairwise amino-acid covariation values $C_{\alpha \beta }^{ij}=f_{\alpha \beta }^{ij}-f_{\alpha }^{i}f_{\beta }^{j}$ [12]. A site-independent model should have zero connected correlation at all orders. In Figs. 4(c) and 4(d), MSA-T scores significantly worsen using cc-*r*_20_ than the Potts model. Interestingly, in Fig. 4(d), where synthetic data were generated by MSA-T, the Potts model does not match the null expectation, possibly indicating that MSA-T introduces statistical patterns beyond pairwise interactions. However, in natural sequence tests (Fig 3), the Potts model matched the null expectation, suggesting such patterns may not exist in natural datasets. MSA-T also tends to generate less variation than the Potts model, explaining why the null result is larger in Fig. 4(c) compared to Fig. 4(d).

## VI. THE POTTS MODEL OUTPERFORMS MSA-T WHEN TRAINED USING PHYLOGENETICALLY REDUNDANT MSAs

**FIG. 5.**
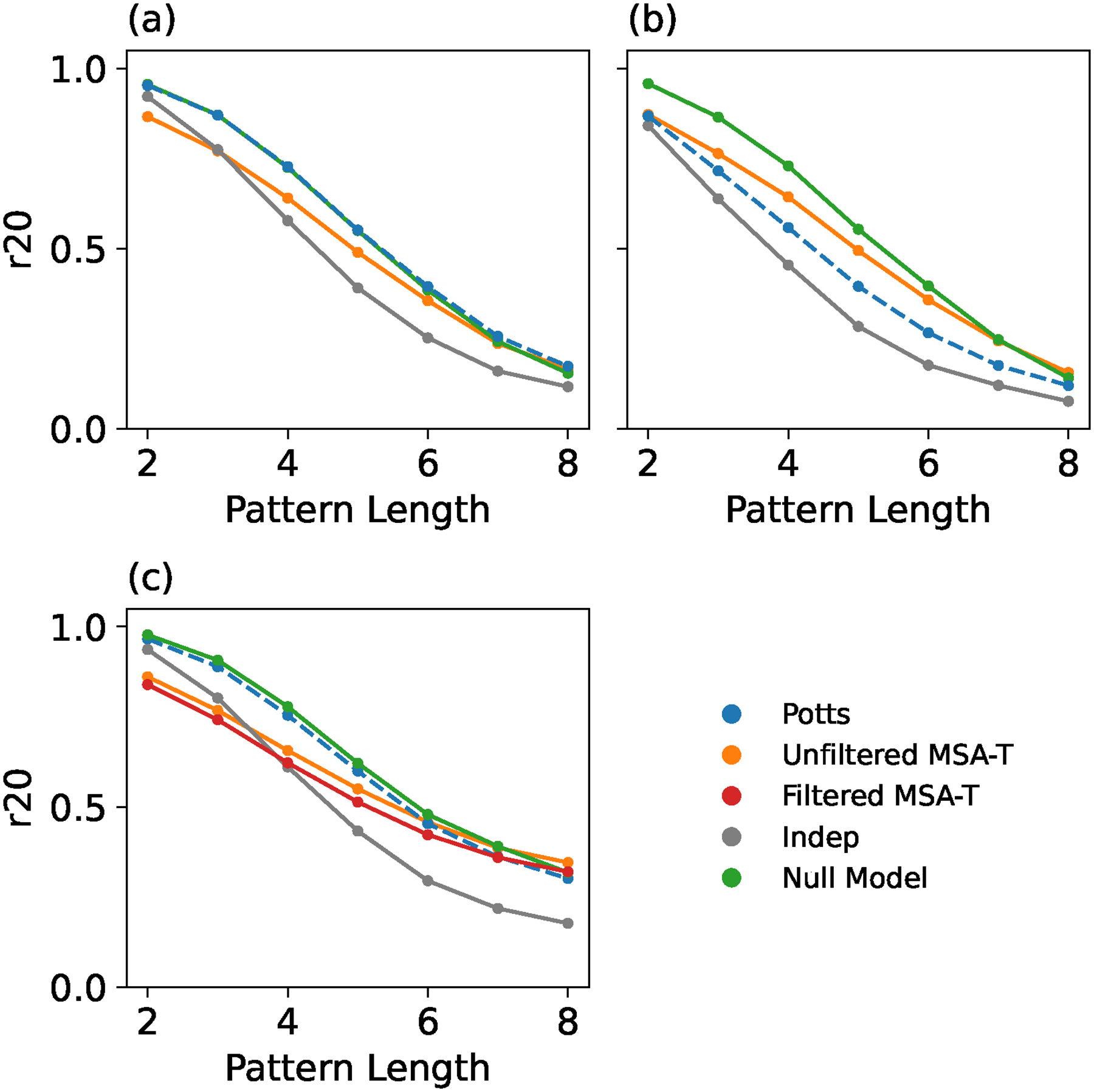
Tests of the impact of identity filtering for the RR-domain family. (a) Models using 30K unfiltered natural sequences for both training and evaluation. (b) The Potts and Independent models trained on 6K filtered natural sequences; MSA-T was trained on 30K unfiltered sequences. The reference MSA is 30K unfiltered. (c) *r*_20_ value for all models, evaluated against 6K filtered natural sequences.

In Ref. [23], it was suggested that MSA-T may have an advantage by being insensitive to phylogenetic structure due to its columnwise attention layers, avoiding the need for identity filtering that has high computational complexity *O*(*N*^2^) for MSA depth *N*. In Ref. [23], MSA-T was trained and evaluated on randomly divided MSAs without identity filtering, and the results showed that it outperformed the Potts model, according to *r*_20_ tested against the unfiltered reference MSA, which we reproduced in Fig. 5(b). However, the Potts model used in that test implicitly performed identity filtering as an internal step. While this can be reasonable if treating the Potts software as a black-box GPSM, it should be expected that a model trained on a filtered MSA will have a lower *r*_20_ when evaluated with unfiltered reference MSA. This suggests to test performance when the Potts model is both trained and evaluated on unfiltered MSAs. We find that the Potts model outperforms MSA-T in this case [Fig. 5(a)], suggesting that the Potts model is able to model the component of correlations caused by phylogenetic clusters, to some degree. We expect that such correlations are not biophysical in origin, so that while intriguing this test is inappropriate for testing which GPSM best captures biophysical constraints.

Instead, we suggest that using filtered MSAs for model evaluation will give the best measure of a GPSM’s ability to capture the underlying biophysical fitness function, as this will minimize the effects of phylogenetic clustering that introduce sequence correlations driven by nonselective parameters like speciation rates and experimental sampling bias, as discussed above. In Fig. 5(c), we compare multiple GPSMs using a filtered reference MSA, and in particular compare MSA-T performance using either filtered or unfiltered training MSAs. MSA-T shows similar performance either way, suggesting that it implicitly corrects for phylogenetic structure in the training MSA, if present. However, when the Potts model is trained using a filtered MSA, it generally outperforms MSA-T and closely matches the null expectation for a well-specified model. This supports the conclusion that the Potts model more accurately captures features of the underlying biophysical fitness function, as measured by *r*_20_, than MSA-T.

In addition to the *r*_20_ analysis, we evaluated simpler one-site statistics for the filtered training MSA and the model-generated MSAs from it, using sequence logos generated by the WebLogo [29,30] tool, and per-site entropy for all models. The Potts and Independent models very closely match the conservation patterns of the natural MSA, while MSA-T exhibits overconservation and lower entropy [see Figs. S3(a), S3(b), and S4 in the Supplemental Material [25]]. These results further support the Potts model’s stronger ability to reproduce the statistical features of the training MSA.

## VII. DISCUSSION

The impact of phylogenetic relationships on GPSMs and protein covariation analysis has been recognized since early Potts studies [31–34]. Various methods have been proposed to address its confounding effects, such as the “average product correction” [35,36] and identity weighting [4], or in profile HMMs used by HMMER [37], which are a form of GPSMs, by weighting like the Henikoff scheme [38]. Spectral decomposition of the pairwise covariation matrix [39], central to Potts inference, shows that its low eigenmodes are influenced by phylogeny, while high eigenmodes capture biophysical mutational couplings due to epistasis, and that Potts inference is insensitive to low eigenmodes. This insensitivity has also been found empirically when predicting biophysical “contacts” in proteins [40]. This suggests that the Potts model may accurately model biophysical interactions even if identity filtering does not completely account for phylogenetic clustering. MSA-T is also designed to account for phylogeny through column attention heads, and it has been found that some attention heads effectively detect sequence relationships [11,21].

These results are consistent with previous studies comparing the ability of Potts models and other GPSMs to capture other aspects of protein data, including contacts in observed three-dimensional structures of proteins [41,42], experimental fitnesses, or fitness changes upon mutation [16,43], which find the architecturally simple Potts model performs favorably. For instance, a previous comparison found that the Potts model outperforms MSA-T for contact prediction if the input data have phylogenetic structure removed [11].

Here, we showed the model’s ability to reproduce higher-order sequence covariation, which is a necessary but not sufficient condition for GPSMs to produce functional, natural-like proteins. However, it has been shown that sequences generated computationally using a Potts model are functional *in vivo* [44], and similarly for other recent generative models [45–47]. Additionally, we observe that sequences generated from the Potts model, MSA-T, and the Independent model are all predicted to fold appropriately by the structure-prediction program AlphaFold [48] (see the Supplemental Material [25]). While this suggests that these sequences are foldable, experimental validation is essential to assess whether the generated sequences are fully functional.

We hypothesize that the Potts model outperforms MSA-T in capturing biophysical constraints because (1) it is trained on a specific protein family, while MSA-T is trained on all families; (2) it is directly trained to reproduce pairwise sequence statistics, whereas MSA-T is trained for a masked learning task and so its predictions of marginals are unsupervised; and (3) the Potts model generates sequences with the same diversity as the training MSA, while MSA-T has a free parameter (“replacement rate”) making unclear which value to choose [23]. The two models capture higher-order correlations in potentially different ways: The Potts model is able to model higher-order covariation through networks and chains of pairwise interactions, which may be sufficient to represent a broad class of higher-order correlations, whereas MSA-T leverages deep-learning network layers with nonlinear activations that can, in principle, capture covariation inaccessible to the Potts model. These findings imply that, despite its architectural simplicity, the Potts model best captures functional and structural protein constraints and highlights the importance of carefully decomposing the origins of covariation, not only when training GPSMs, but also during evaluation, and in their practical use for understanding the protein biophysics.

## Supplementary Material

Supplemental Text

## Data Availability

For our study, the RR-domain and kinase MSAs were constructed from the PF00072 and PF00069 seed MSAs from the Pfam database [49], respectively. We used the Mi3-GPU software package to generate the synthetic sequences from the Potts model in our study, which is publicly accessible via its GitHub repository: [50]. To generate the synthetic sequences using MSA-T, we used the publicly available package available via GitHub at [51], which is also archived at Zenodo: [52]. Furthermore, the necessary scripts, data, and files to reproduce the results reported in our study are provided in the publicly accessible Zenodo repository: [53] and on GitHub: [54].

## APPENDIX: EXTENDED METHODS

### 1. Natural MSA dataset preparation

For the RR-domain and protein-kinase families, we constructed MSAs using HHblits [55] to search the Uniclust database version 2023_02 [56]. For RR domain, we used the PF00072 seed MSA from Pfam database [49]. For kinase, we used the PF00069 seed MSA from Pfam [49], with pseudogenes removed as performed in Ref. [12], resulting in 73 062 raw sequences for RR domain and 291 731 raw sequences for kKinase.

### 2. Natural MSA preprocessing

We used identity filtering with a 50% identity threshold, resulting in MSAs of 12 906 sequences for RR domain and 20 000 sequences for kinase. The RR domain was split into 6000 sequence training and reference MSAs, and the kinase into 10 000 sequence training and reference MSAs.

When dividing unfiltered natural MSAs into training and reference sets, there can be statistical dependencies that may lead to underestimating out-of-sample error and overestimating *r*_20_. To address this, we filter the MSA at a 50% identity threshold before splitting into training and reference halves. We then assign each unfiltered sequence in the original natural MSA to either training or reference set, depending on most similar sequence, ensuring closely related sequences are grouped together. This procedure minimally affects *r*_20_.

### 3. Model training

For MSA-T, we tested both “standard” and “alternative” sequence generation methods from Ref. [23], with some modifications.

In the standard method, we divided the input MSA into 600 sequence batches and iteratively performed the masked MSA prediction task for 200 rounds per batch with a masking rate of *p* = 0.1 and default options, using the “greedy” sampling strategy. Sequences with uninitialized characters (〈*cls*〉) were discarded, and new sequences were regenerated to consist only of amino acid characters or gaps. Although convergence measures plateaued after 200 rounds, as noted in Ref. [23], continued iteration led to an accumulation of “gap” characters, especially at the sequence ends, which pronounced more at higher *p* values. We attribute this artifact in MSA-T predictions to missing terminal sequences in the training data, due to shotgun sequencing, bioinformatics misannotation, or evolutionary length variation in protein termini. To address this effect biasing the higher-order correlations, we modified the iterative masking procedure so that gap characters were never masked and never generated at masked positions, preserving the input MSA’s gap characters. We computed bivariate marginals of input MSA with a pseudocount as described in Ref. [24] of scale 1/*N*, where *N* is the input MSA depth. For training of the Potts model, we used the Mi3-GPU inference software [24].

In the “alternative” generation procedure, each sequence is iterated separately with 599 randomly drawn sequences from the input MSA, using the “logits” masked sampling strategy. We modified this to preserve gap structure and fix issues where masked positions were treated as unmasked.

### 4. Higher-order marginal

We use the HOM_r20 package [12] to calculate the precision of the model in reconstructing the higher-order marginals (HOMs) of MSA. We calculate the *r*_20_ value [12] for each HOM of the second to eighth order using 3000 randomly selected column sets of natural and evaluation MSAs. We compute the 20 most frequent amino acid subsequences for each position in training and evaluation MSAs, and then calculate the Pearson correlation (*r*) between these frequencies. The average *r*, called the *r*_20_ metric, indicates how well the generated MSA reconstructs the sequence statistics and higher-order mutational patterns of the natural MSA.

### 5. Synthetic MSA generation and analysis

We generate “synthetic” evaluation MSA datasets using models generatively. First, with the protein-kinase Potts model fit to the 12.9K filtered MSA of the RR Domain, we created 6M “synthetic training” MSAs and two sets of 6M “synthetic reference” MSAs, which were used to construct the null model, using Mi3-GPU via Markov Chain Monte Carlo (MCMC), an algorithm used to draw samples from a given probability distribution [24]. We then trained a new Potts model, as well as MSA Transformer (as implemented in Ref. [23]) and Independent models on the 6M synthetic training MSA and generated synthetic “evaluation” MSAs containing 6M sequences for all models. We repeated a similar process for the MSA Transformer using the same input as shown in Fig. 2. This approach addresses finite sampling limitations by generating MSAs with any desired number of sequences.
