# Supplementary material for: Phylogenetic corrections and higher-order sequence statistics in protein families: Potts vs multiple sequence alignment transformer machine learning models
Source: Phys Rev Res. Author manuscript; Available in PMC 2026 Jun 11. (PMC13251796; doi:10.1103/g5cx-1vhj)
Supplement: Supplemental Text [file NIHMS2179467-supplement-Supplemental_Text.pdf]

Supplemental Material: Phylogenetic Corrections and Higher-Order Sequence Statistics in Protein Families: The Potts vs MSA Transformer Machine Learning Models

Kisan Khatri,[1] Ronald M. Levy,[2] and Allan Haldane[3,*]

[1]Department of Physics and Center for Biophysics and Computational Biology, Temple University, Philadelphia, PA 19122, USA

[2] Department of Chemistry and Center for Biophysics and Computational Biology, Temple University, Philadelphia, PA 19122, USA

[3] Department of Physics and Center for Biophysics and Computational Biology, Temple University, Philadelphia, PA 19122, USA

Two Body Connected Correlation

To evaluate how well each model reproduces the pairwise residue covariation in the training multiple sequence alignment (MSA), we computed the two-body connected correlation from the filtered training MSA and from MSAs generated by the Potts model and the MSA Transformer (MSA-T). This second-order connected correlation is defined as:

$$C^{ij}_{\alpha\beta} = f^{ij}_{\alpha\beta} - f^i_\alpha f^j_\beta \tag{1}$$

Where $f^{ij}_{\alpha\beta}$ is the joint frequency of amino acids α and β at positions i and j, respectively, and f^i_α and f^j_β are the single-site frequencies of residues α at position i and β at position j, respectively[1].

To assess the internal consistency, we also include the null model by splitting the filtered natural MSA into two halves and compared the two body statistics between them. The Pearson correlation coefficient for each model is reported which quantifies the agreement between the connected correlations computed from the model-generated MSAs and the natural MSA. The null model provides a baseline, where deviation from it reflects reduced modeling accuracy. The Potts model shows stronger agreement with the natural MSA (r = 0.768) than the MSA-T (r = 0.501), supporting our finding that the Potts model more accurately captures the covariation structure of the MSA than MSA-T, as shown in Figure SM1.

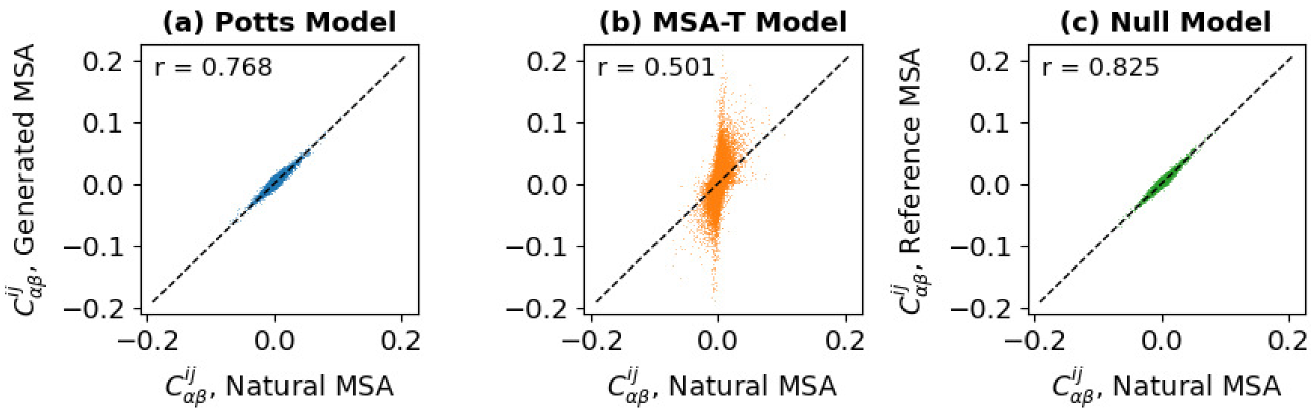

Figure SM1. Comparison of two-body connected correlations estimated from the generated MSAs and the natural MSA for RR Domain (PF00072).

Foldability of Model-Generated Sequences

To assess the foldability of the model-generated sequences, we selected five representative sequences from each of the Potts model and MSA-T, and the Independent model-generated MSAs, generated from the same input filtered

MSA (6K) of the RR domain protein. We used AlphaFold2 (AF2) via the ColabFold implementation[2] to predict the 3D structures of each sequence and structurally aligned them to the crystal structure of the RR domain (PDB ID: 3ILH) using PyMol[3]. All predicted structures exhibited close alignment as shown in Figure SM2, suggesting that sequences generated by all models retain the ability to fold into a native-like structure. The average RMSD values with respect to the crystal structure were 2.36 Å (Potts), 2.54 Å (MSA-T), and 2.66 Å (independent). A recent study in [4] showed that RMSD values from AF2 predictions are quantitatively correlated with protein foldability, with lower RMSD values indicating greater foldability. Our results support this trend and further suggest that Potts and MSA-T generated sequences may capture biophysical constraints important for foldability better than sequences from the independent model, as we can see the AF2 predicted structures for the Potts model and MSA-T seem to be more properly aligned than the Independent model. However, it has also been mentioned that RMSD values of computational models are not quantitatively correlated to the protein function[2].

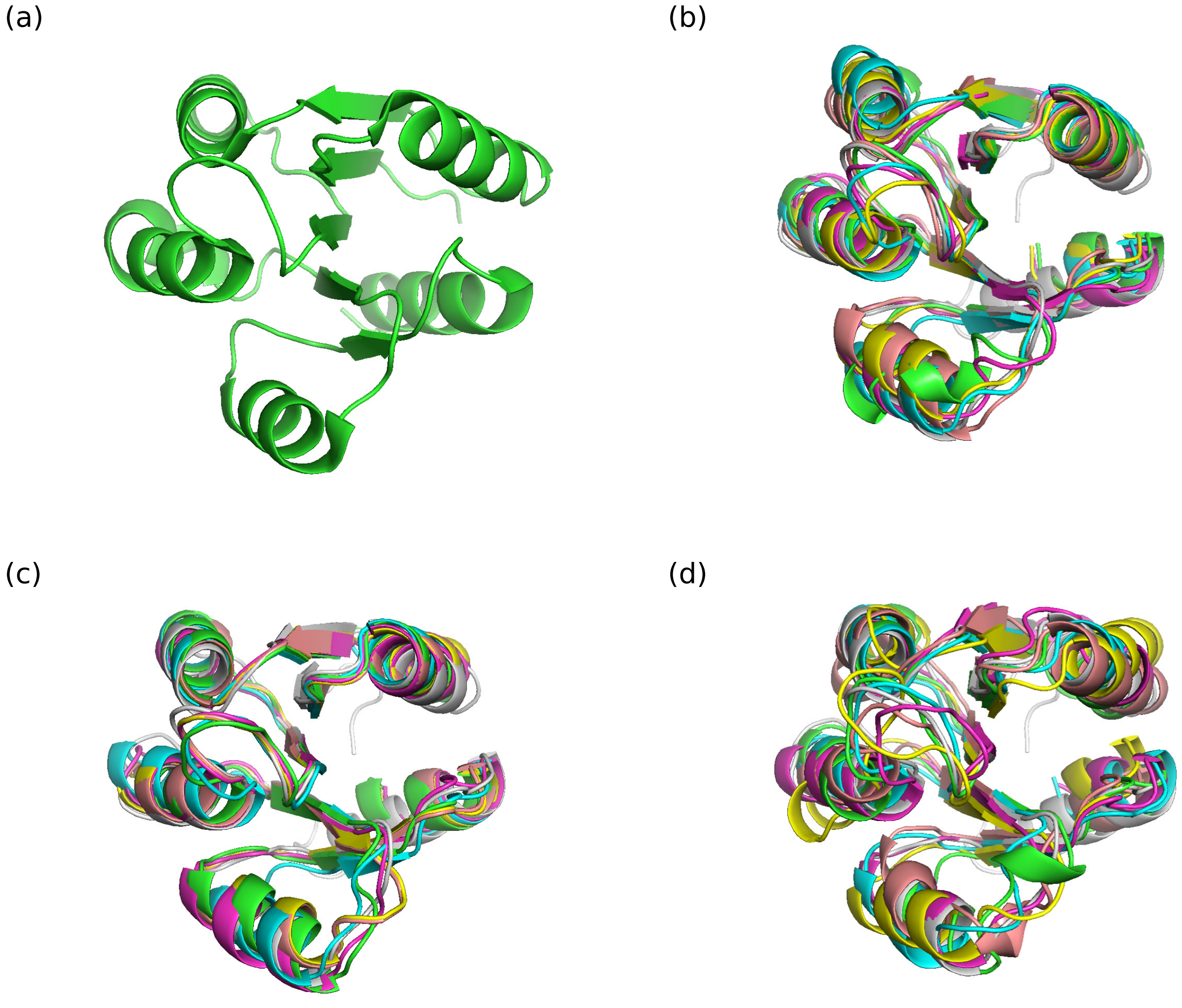

Figure SM2. Testing the foldability of model-generated sequences. (a) Crystal structure of the RR domain (PDB ID: 3ILH). (b), (c), and (d) AlphaFold2 (AF2) predicted structures aligned with the crystal structure for sequences generated by the Potts model, MSA Transformer (MSA-T), and the independent model, respectively. The predicted structures show close alignment with the crystal structure. The average RMSD values with respect to the crystal structure are 2.36 Å, 2.54 Å, and 2.66 Å for the Potts, MSA-T, and independent models, respectively.

Site Conservation and Variation Pattern of the MSA

We used WebLogo[5, 6], an online-based tool which creates the sequence logos to visualize the patterns in the MSAs, which displays a clear representation of how conserved or variable each residue position within the aligned sequence, where each stack of amino acid letters indicates its frequency at that position. The height of each letter within a stack is proportional to its observed frequency, making it easy to interpret conservation patterns across alignment. We generated sequence logos for the filtered natural MSA (6K sequences) and the model-generated MSAs (6K sequences each) from three models. The results are shown in Figure SM3-(a) and Figure SM3-(b). The sequence logos produced by the Potts model and the independent model exhibit similar patterns to those of the natural MSA. Residues at corresponding positions display comparable frequencies and distributions. This aligns with the design of the Potts model, which is trained to match both single-site and pairwise amino acid frequencies of the input MSA, further supporting its effectiveness. In contrast, the sequence logo generated by the MSA Transformer (MSA-T) differs noticeably from that of the natural MSA. It shows higher conservation at many positions, suggesting over-representation of dominant residues. This deviation is consistent with our observations from the two-body connected correlation analysis discussed in Supplementary Information SM1.

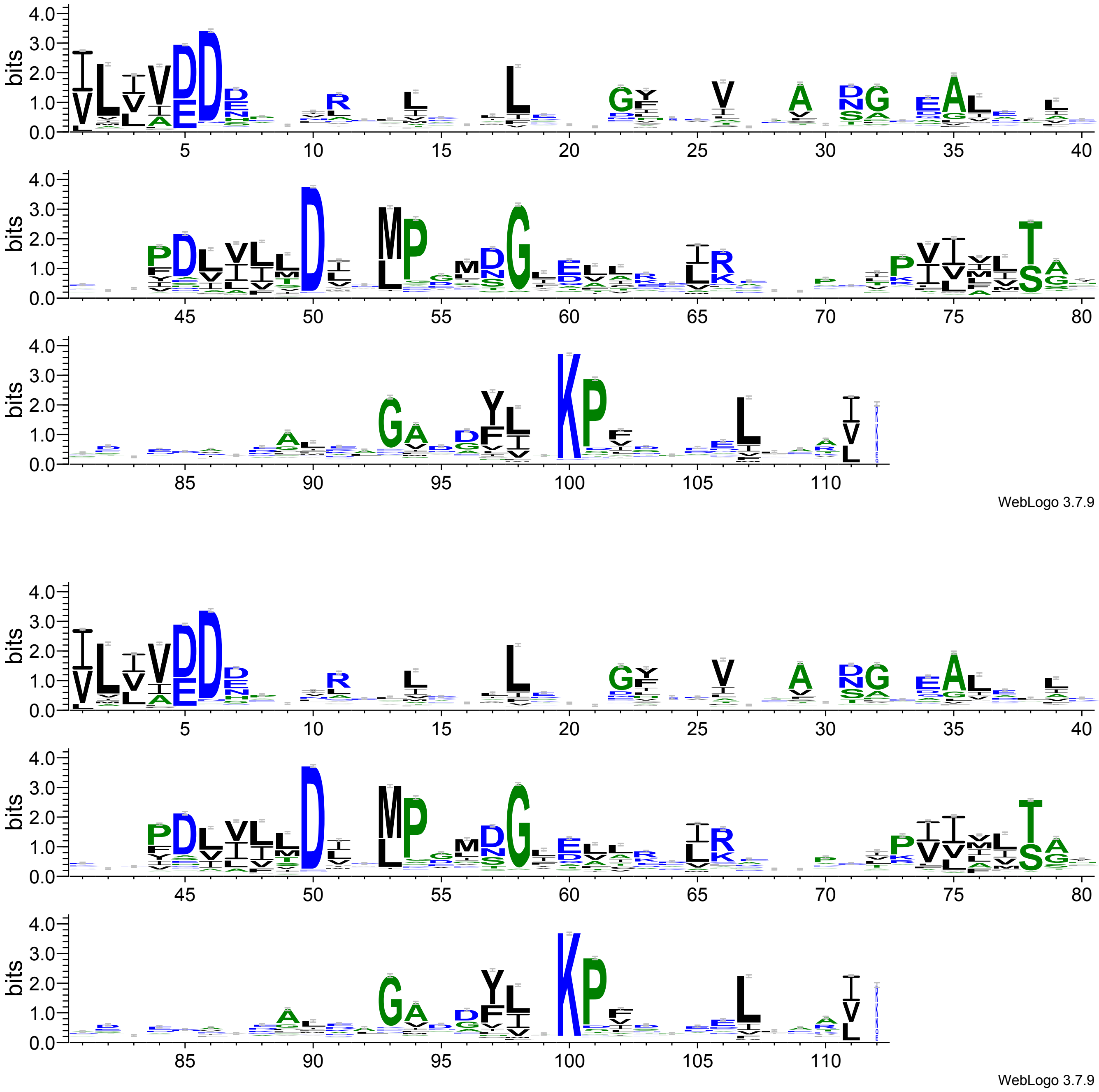

Figure SM3-(a). Sequence logos for the filtered natural MSA (top) and the Potts model-generated MSA (bottom), each with 6K sequences, for the RR domain. Potts model closely matches the natural MSA.

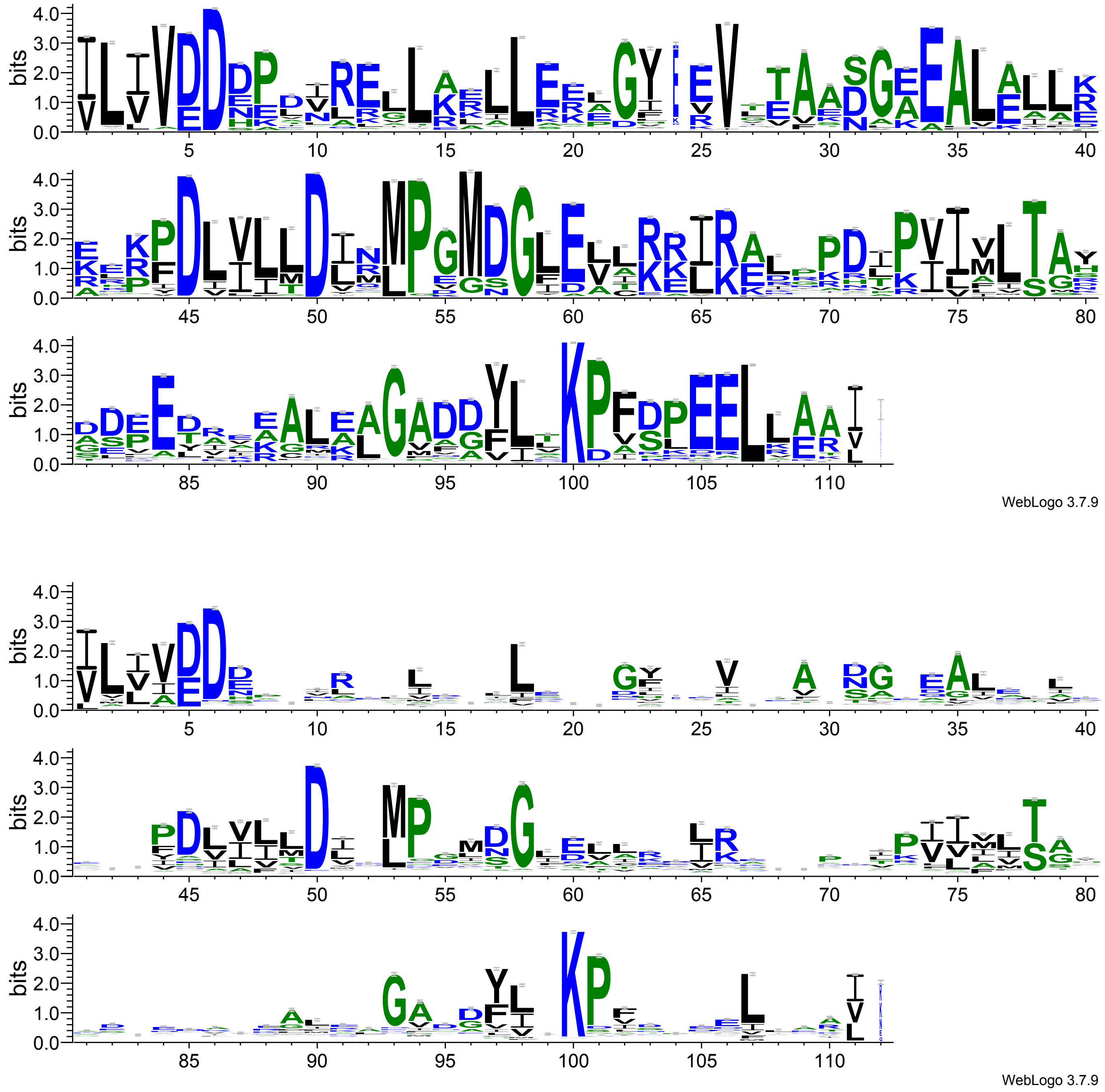

Figure SM3-(b). Sequence logos for the MSA-T (top) and the Independent model-generated MSAs (bottom), each with 6K sequences, for the RR domain. The Independent model closely matches the natural MSA, while MSA-T shows over-conservation.

Entropy/Single Site Conservation

Further, we have also presented the results of the entropy/single-site conservation analysis for the RR domain. These results show that MSA-T generates sequences with lower per-site entropy than the training MSA, the Potts model, and the independent model, whose per-position entropy and per-site conservation are closely aligned. This indicates that MSA-T puts more emphasis on conserved sites, i.e., low-entropy sites. Particularly, the Potts model preserves site-wise entropy across the training MSA, effectively capturing the diversity and balance of conservation in actual sequences. These results underscore the Potts model's stronger ability to accurately reproduce the statistical features of the training MSA compared to the MSA Transformer.

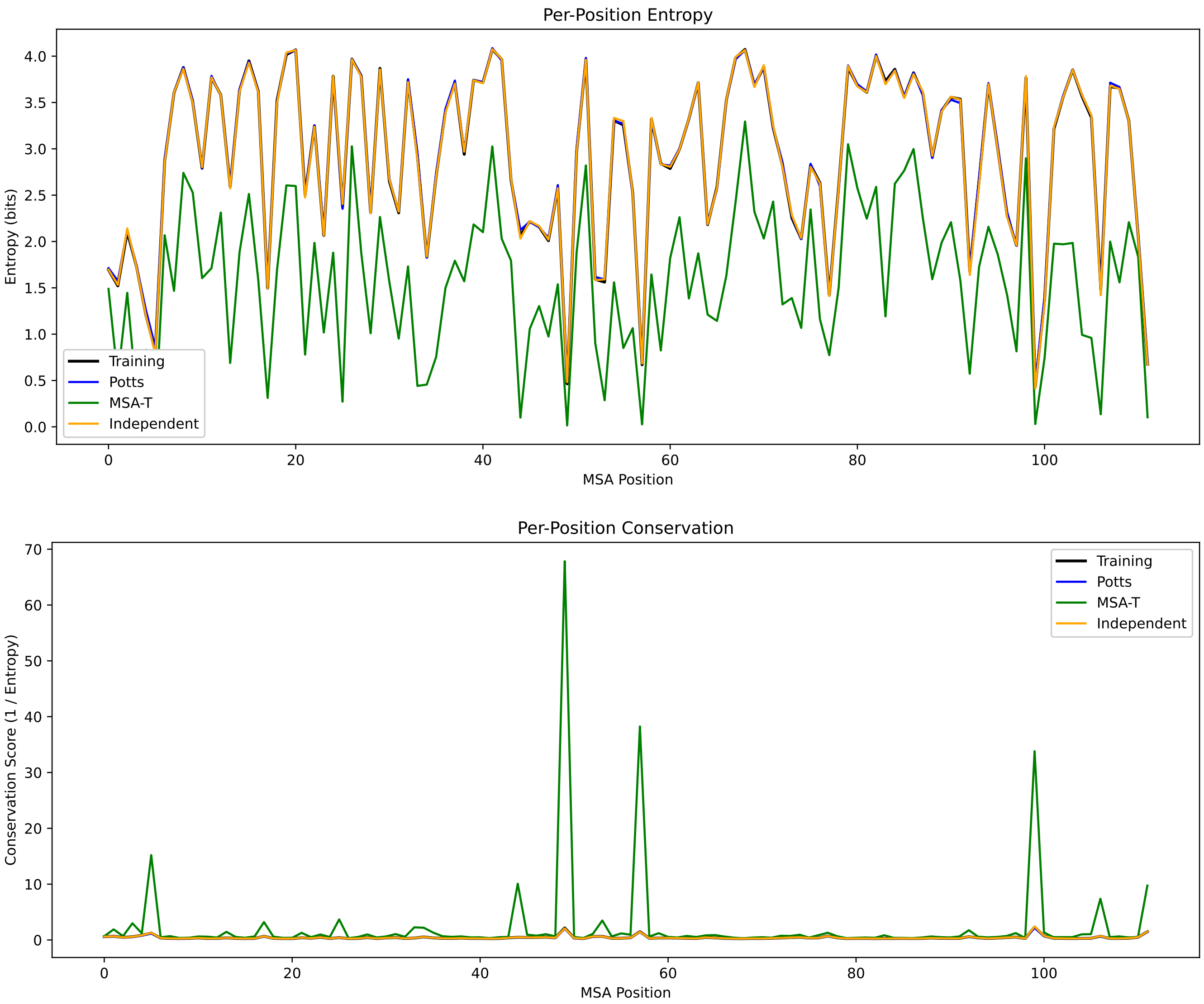

Figure SM4. Per-site entropy (top)/conservation (bottom) comparison between the filtered natural MSA and MSAs generated by the Potts model, MSA Transformer (MSA-T), and independent model, each containing 6K sequences, for the RR domain. The Potts and independent models closely match the natural MSAs' site-wise entropy, capturing sequence diversity and conservation balance, while MSA-T produces sequences with systematically lower entropy, emphasizing conserved sites.

Together, the two-body correlation analysis, site conservation assessments (sequence logos and per-position entropy), and foldability evaluations demonstrate that the Potts model more accurately captures the statistical and structural properties of the natural MSA than the MSA Transformer. Notably, sequences generated by the Potts model exhibit better foldability, with structures aligning more closely to the native fold, showing the lowest RMSD among the three models.

* Electronic address: allan.haldane@temple.edu

[1] D. Sgarbossa, U. Lupo, and A.-F. Bitbol, Elife **12**, e79854 (2023).
[2] M. Mirdita, K. Schütze, Y. Moriwaki, L. Heo, S. Ovchinnikov, and M. Steinegger, Nature methods **19**, 679 (2022).
[3] W. L. DeLano, http://www. pymol. org/ (2002).
[4] S. Liu, K. Wu, and C. Chen, Computational and Structural Biotechnology Journal **20**, 4481 (2022).
[5] T. D. Schneider and R. M. Stephens, Nucleic acids research **18**, 6097 (1990).
[6] G. E. Crooks, G. Hon, J.-M. Chandonia, and S. E. Brenner, Genome research **14**, 1188 (2004).
